# Modulating microbiome-immune axis in the deployment-related chronic diseases of Veterans: report of an expert meeting

**DOI:** 10.1080/19490976.2023.2267180

**Published:** 2023-10-16

**Authors:** Jun Sun, M. Nedim Ince, Clara Abraham, Terrence Barrett, Lisa A. Brenner, Yingzi Cong, Reza Dashti, Pradeep K. Dudeja, David Elliott, Thomas S. Griffith, Peter S. Heeger, Andrew Hoisington, Kaikobad Irani, Tae Kon Kim, Neeraj Kapur, Joseph Leventhal, Mansour Mohamadzadeh, Ece Mutlu, Rodney Newberry, Jonathan U. Peled, Israel Rubinstein, Salyka Sengsayadeth, Chen Sabrina Tan, Xiao-Di Tan, Eric Tkaczyk, Jason Wertheim, Zheng Jenny Zhang

**Affiliations:** a Jesse Brown Veterans Affairs Medical Center, Chicago, IL, USA; b Division of Gastroenterology and Hepatology, Departments of Medicine, Microbiology/Immunology, University of Illinois Chicago, Chicago, IL, USA; c Iowa City Veterans Affairs Medical Center, Lowa city, IA, USA; d Division of Gastroenterology and Hepatology, Department of Internal Medicine, University of Iowa, Iowa City, IA, USA; e Medicine, Yale University, New Haven, CT, USA; f Lexington Veterans Affairs Medical Center Kentucky, Lexington, KY, USA; g Medicine, University of Kentucky, Lexington, KY, USA; h Veterans Affairs Rocky Mountain Mental Illness Research, Education, and Clinical Center, Aurora, CO, USA; i Physical Medicine and Rehabilitation, University of Colorado, Anschutz Medical Campus, Aurora, CO, USA; j Microbiology and Immunology, University of Texas Medical Branch at Galveston, Galveston, TX, USA; k Medicine, Stony Brook University, Stony Brook, NY, USA; l Minneapolis VA Medical Center, Minneapolis, MN, USA; m Urology, University of Minnesota, Minneapolis, MN, USA; n Medicine/Nephrology, Cedars-Sinai Medical Center in Los Angeles, Los Angeles, CA, USA; o Tennessee Valley Healthcare System-Nashville VA, Nashville, TN, USA; p Vanderbilt University, Nashville, TN, USA; q Surgery, Northwestern University, Evanston, IL, USA; r Microbiology, University of Texas Health Science Center at San Antonio, USA, TX, San Antonio; s Washington University in Saint Louis School of Medicine, St. Louis, MO, USA; t Adult Bone Marrow Transplantation Service Memorial Sloan Kettering Cancer Center, New York, NY, USA; u Surgery, University of Arizona, Tucson, AZ, USA; v Tucson VA Medical Center, Tucson, AZ, USA

**Keywords:** Immunity, IBD, infection, gut-brain-axis, graft-versus-host disease, oral microbiome, graft rejection, posttraumatic stress disorder, gut microbiome, mitochondria, micronutrition, vitamin, virome

## Abstract

The present report summarizes the United States Department of Veterans Affairs (VA) field-based meeting titled “Modulating microbiome-immune axis in the deployment-related chronic diseases of Veterans.” Our Veteran patient population experiences a high incidence of service-related chronic physical and mental health problems, such as infection, irritable bowel syndrome (IBS), inflammatory bowel disease (IBD), various forms of hematological and non-hematological malignancies, neurologic conditions, end-stage organ failure, requiring transplantation, and posttraumatic stress disorder (PTSD). We report the views of a group of scientists who focus on the current state of scientific knowledge elucidating the mechanisms underlying the aforementioned disorders, novel therapeutic targets, and development of new approaches for clinical intervention. In conclusion, we dovetailed on four research areas of interest: 1) microbiome interaction with immune cells after hematopoietic cell and/or solid organ transplantation, graft-versus-host disease (GVHD) and graft rejection, 2) intestinal inflammation and its modification in IBD and cancer, 3) microbiome-neuron-immunity interplay in mental and physical health, and 4) microbiome-micronutrient-immune interactions during homeostasis and infectious diseases. At this VA field-based meeting, we proposed to explore a multi-disciplinary, multi-institutional, collaborative strategy to initiate a roadmap, specifically focusing on host microbiome-immune interactions among those with service-related chronic diseases to potentially identify novel and translatable therapeutic targets.

## Meeting background

The Department of Veterans Affairs (VA) Field-Based meeting, which took place in Chicago on May 5, 2023, was organized to facilitate discussions on microbiota-immune interactions in both homeostasis and disease, with the goal of potentially developing a multidisciplinary research collaboration in which state-of-the-art approaches would be used to investigate deployment-related chronic diseases affecting Veterans. Specific investigators and potential collaborators from the VA-funded space were invited to share novel ideas and visions related to foregoing topics, which are expected to initiate fruitful collaborations.

Members of our Veteran patient population experience a high incidence of service-related chronic illnesses. These include infections, irritable bowel syndrome (IBS), inflammatory bowel disease (IBD), various forms of hematological and non-hematological malignancies, end-stage organ failure requiring transplantation, and mental health and neurologic disorders associated with the gut-brain axis, including posttraumatic stress disorder (PTSD). Many of these diseases can be triggered by severe stress, exposure to environmental toxins (e.g., agent orange), and altered composition of the microbiome. The human microbiome includes bacteria, viruses, and fungi that reside in our body. In the meeting, we emphasized the role of the microbiome not only in the intestine, but also in other organs, such as mouth, nose, lung, blood, and tumor. Environmental exposures, e.g., burn pit, jet fuel, may occur while individuals are deployed to diverse geographies nearby or overseas, particularly in the context of combat conditions. The altered composition and function of the gut microbiome and associated metabolites in deployed military personnel may also occur in areas with suboptimal hygienic conditions. The service connections of many of these disorders have been extensively investigated and established, particularly since the Gulf War. Previous studies have documented links between service-related conditions and environmental factors, including the causative link between agent orange exposure and hematological malignancies in Vietnam War Veterans. These service-related disorders can be lethal (e.g., cancer), and many of them are painful, disabling, and chronic. Long after deployment, service members can experience ongoing symptoms and disease sequelae, demonstrate organ failure, or develop malignant disorders that require either hematopoietic cell transplantation or solid organ transplantation. Transplantation can result in lethal and devastating graft-versus-host disease or graft rejection.

**Figure 1.**
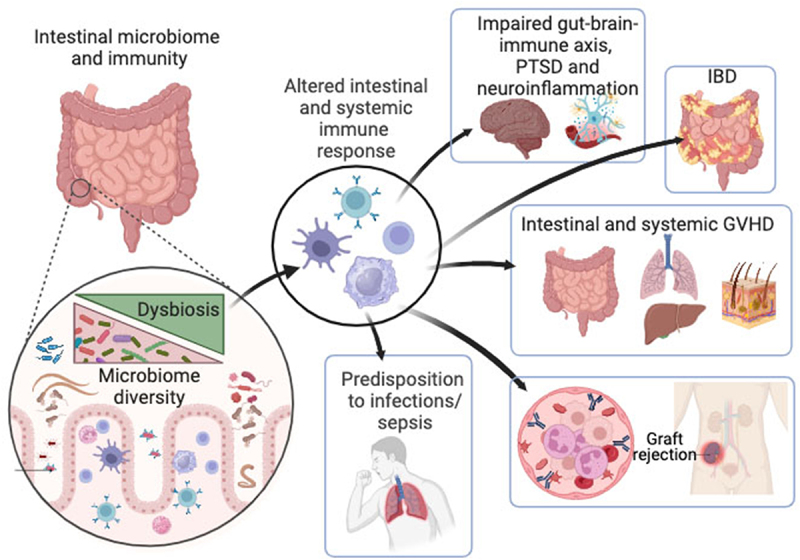
Impact of microbiome-immune axis on intestine and peripheral physiologic homeostasis.

Recent scientific discoveries have greatly enhanced our understanding of the complex crosstalk between microbiome and host immunity. Altered composition and function of the microbiome can lead to various disorders. Altered microbiome composition and metabolic activities thereof, also called dysbiosis not only affects intestinal homeostasis but also extraintestinal organs, including the brain, lung, liver, and skin (Figure 1). Dysregulation of microbiome-immune interactions may manifest in various organ pathologies, including IBD, cancer, and neuroinflammatory diseases. Crosstalk between the microbiome and immune cells can also affect the outcome of treatment, as observed in Veterans undergoing hematopoietic cell transplantation (HCT) to treat hematological malignancies, where gut dysbiosis is highly associated with the development of lethal and devastating GVHD. Many unknowns and challenges remain to be elucidated by disentangling the underlying mechanisms involving microbiome-immunity interplay in hosts during homeostasis and disease.

## Focused topics

The goals of our VA Field-Based meeting were to further define the significance of microbiota and associated metabolites that drive the fate of host immunity and to collaboratively elucidate potentially involved mechanisms in the microbiome and associated metabolites in both, health and disease, and the consequences thereafter. To identify fruitful research targets and promote collaboration between VA-based researchers/clinicians and non-VA affiliated researchers, short talks/idea exchanges were presented that centered on the areas specified below. Such collaborative efforts are expected to utilize multiomic approaches, apply machine learning, establish novel genetically engineered animal models, and employ organoids from biopsies of Veterans to interrogate mechanisms implicated in diseases and the potential link to microbiome–host interactions. Furthermore, the meeting highlighted various aspects of the current knowledge gaps and discussed the molecular mechanisms potentially driving local and peripheral disease progression. Moreover, at the conference, investigators vividly discussed the dysregulation of microbiome-immune interactions in various human diseases, and the challenges and limitations in achieving a causal understanding of host microbiome-immune interplay to obtain critical insights into these areas of interest, which can translate toward future development of microbiome-targeted therapeutic interventions. The focus was on novel and personalized management possibilities for these systemic inflammatory diseases. These disorders are grouped in 4 focus areas and include:

### Focus 1: microbiome interactions with the immune system after hematopoietic cell and/or solid organ transplantation (GVHD and graft rejection)

Each year, more than 20,000 hematopoietic cells and more than 35,000 solid organ transplantations are performed in the United States. Hematopoietic cell transplantation (HCT) is performed in most cases as a curative treatment for hematological malignancies, such as leukemia or myeloma, whereas solid organ transplantation (SOT) is a state-of-the-art therapeutic practice to restore the function of a failing organ, such as the kidney, liver, or heart. In certain cases, the combined transplantation of hematopoietic cells and solid organs is performed to treat hematological malignancies and organ failure.

Veterans constitute an important group of these patients because they are prone to develop hematological malignancies due to exposure to different toxins during their service, such as agent orange. Veterans are more likely to develop end-stage organ failure. The frequency of kidney disease was higher among Veterans. Furthermore, more than 40,000 Veterans with end-stage renal disease are enrolled in the VA Health System and receive treatment via dialysis or transplantation. The outcome of transplantation is adversely affected by graft-versus-host disease (GVHD) after HCT, and organ rejection after SOT. In this context, studies suggest that intestinal microbiota is dysregulated after HCT and in patients who develop GVHD,^1^ and monitoring and remodeling of microbiota after HCT can lead to the development of novel diagnostic tools, such as new disease markers or novel therapeutics in the form of elements of gut microbiota.^2^ Similarly, dysbiosis occurs after SOT^3^, and it is believed to be caused by immunosuppressive therapy, which while preventing rejection, predisposes the host to various complications, such as life-threatening infections, cancer, or metabolic syndrome. Achieving immune tolerance after solid organ transplantation without the use of immunosuppressive medications is an ideal endpoint in clinical transplantation and is being investigated by inducing in vivo tolerogenic states, such as mixed chimerism.^4^ Whether targeting intestinal microbiota modulates graft rejection is an important question in transplantation medicine,^5^ which the Focus 1 group asked during the Field-Based Meeting in an attempt to synergize research efforts in collaboration with other focus groups.

### Focus 2: intestinal inflammation and immune dysfunction (IBD and cancer therapy)

**Figure 2.**
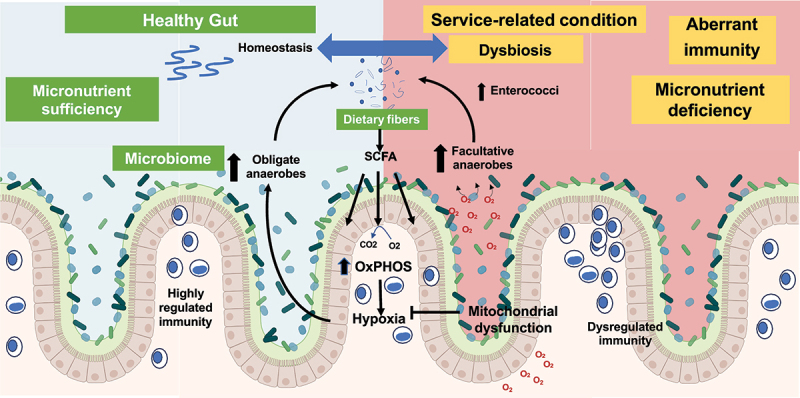
Mechanism of intestinal microbial architecture and immunity in health and disease.

Research into the pathogenesis of IBD has extended across broad landscapes to identify important insights into the roles of the intestinal microbiome and mucosal metabolism in the regulation of intestinal adaptive and innate immune responses. The data presented at this meeting illustrate the role of intestinal epithelial mitochondrial respiration (reduced in IBD)^6^ on barrier function as well as the composition of the intestinal microbiome^7–9^ (Figure 2). Further data highlighted the role of goblet cell-associated antigen passages (GAPs) that deliver luminal substances to the underlying lamina propria (LP) antigen-presenting cells (APCs). A dysbiotic microbiome adversely affects GAP responses needed to maintain regulation of mucosal T cell populations.^10^ The importance of genetic dysregulation of innate regulatory pathways in IBD pathogenesis was highlighted^11^ in the discussion. Ranjan et al. identified IBD-associated genetic variants that confer disease risk through both excessive and inadequate innate immune responses. This work shows the utility of defining the functional implications of pathways identified through genetic associations as a means of understanding disturbances in immune regulation underlying disease activity in patients with IBD.^11^ Using a powerful tool, Focus Group 2 also presented results from a T cell receptor transgenic mouse that was specific for the CBir flagellin antigen. These results indicate that restoration of a healthy microbiome may improve wound healing by enhancing short chain fatty acid (SCFA) induction of IL-22 production.^12^ Overall, a multilayered approach to IBD pathogenesis has raised a multitude of potential targets for novel therapeutic discovery. A similar multilayered approach could be applied to research on colitis-associated colon cancer and other chronic diseases, as discussed in other focus groups. A working model of the microbiome and immunity has been proposed for mechanistic studies (Figure 2).

In healthy individuals, intestines are lined with differentiated intestinal epithelial cells (IECs) that have relatively high levels of mitochondria that consume oxygen (O2) by oxidative phosphorylation (OXPHOS) creating a hypoxic microenvironment. Oxygen consumption reduces O2 availability to the microbiome and promotes a healthy anaerobic environment dominated by obligate anaerobes (commensals). These obligate anaerobes ferment dietary fibers and produce short chain fatty acids (SCFA) that in turn provide substrate for IEC OXPHOS. Besides, the gut microbiota can communicate directly or indirectly to the brain via microbe-derived neuroactive or gut-derived molecules, regulating autonomic nervous system influences that in turn modulate the gut microbial environment. In a healthy gut, microbiome induce naïve CD4+ T-cell differentiation into Th1, Th2, Tregs, and Th17 sub-types, which work together systematically to regulate immune responses, eliminate intracellular pathogens and control infections. Altogether, these factors under sufficient micronutrient bioavailability, synergistically limit gut- and neuro-inflammation and maintain healthy homeostasis. Intestinal inflammation-induced damage impairs mitochondrial function, thereby limiting OXPHOS and converting IECs to a glycolytic metabolism that consumes less O2. Reduced IEC O2 consumption increases the amount of O2 emanating from the colonic epithelium. Increased available O2 promotes expansion of facultative anaerobes that do not produce SCFA. Overall, dysbiosis during chronic disease creates microenvironment that not only lacks energy substrates to fuel IECs but also dysregulates systemic immunity.

### Focus 3: microbiome-neuron-immunity axis (mental and physical health of Veterans)

Owing to genetic and environmental factors, imbalances in microbiota-immune interactions in a susceptible host can trigger severe inflammation and initiate acute and chronic disorders. In this context, neurological and psychiatric disorders are associated with specific molecular and microbial profiles.^13–18^ In the case of posttraumatic stress disorder (PTSD), which can occur after a life-threatening experience, such as combat on the battle field. Individuals develop symptoms, which include severe anxiety, cognitive problems, and challenges with functioning due to psychological distress. Although often conceptualized as a disorder primarily associated with the brain, recent research suggests that PTSD is associated with dysbiosis of the gut and oral microbiomes.^19^ While the cause and effect remain unproven, the relationship between PTSD and the gut microbiome is likely to be bi-directional: alterations in commensal bacteria may modulate PTSD symptoms via gut-derived neurohormonal and immune pathways, while at the same time, trauma and stressor exposure-associated dysautonomia, through the enteric nervous system, may affect gut microbial composition.^20^ Besides the PTSD, military Veterans, regardless of the branch of service, the era in which they served, or whether they served during a time of peace or war, are at a greater risk of succumbing to death from the devastating motor neuron disease, amyotrophic lateral sclerosis (ALS) than if they had not served in the military. For reasons yet unknown, Veterans are twice as likely to be diagnosed with ALS compared to the general population. A study by Martin *et al*. highlighted the novel role of the microbiome in the development and progression of ALS.^18^ Targeting the intestinal microbiome and immunity could be a novel strategy for ALS.^18,21^ As such, the gut microbiome-immune axis constituted the focus of our Field-Based meeting. Specifically, we discussed biological phenomena associated with conditions of interest, as well as the mechanisms underlying these disorders, with the goal of identifying novel therapeutic targets and developing new approaches for clinical intervention. Some work in this area focused on addressing symptoms associated with mild traumatic brain injury and/or PTSD, which can improve after administration of immunomodulatory probiotics, and these studies have already commenced^15^ and were presented. This Focus Group identified a gap in the mechanistic studies of microbiome dysbiosis and immune dysfunction in Veterans affected by PTSD (undernutrition, neuroinflammation, infections).^15–17^ A key direction of these collaborative efforts is to investigate the pivotal role of dysbiosis in modulating the interaction between gut and brain immunity, the impact of induced life-threatening stress, and stress mediators, which potentially ravage barrier integrity and neuronal inflammation. These efforts are expected to facilitate the development of a roadmap to design novel research projects and at the end to provide much-needed strategies or therapeutic approaches, which can promote the homeostatic effects of microbes on host microbiome-immune axis. Same strategies are also anticipated to materialize in microbiome transplant experiments in humans and rodents, including germ-free and gnotobiotic animals.

### Focus 4: microbe-immune-micronutrition interactions in infection and inflammation

This group discussed the critical micronutrients that reprogram the functions of phagocytic cells to resist pathogen-induced inflammation. Multilayered mechanisms implicated in neuroinflammatory diseases such as Alzheimer’s disease (AD) require further investigation. However, tauopathy-associated neuroinflammation can be increasingly induced by risk factors, including micronutrient deficiencies or pathogen infection.^22–32^ Induced pathogenic neuroinflammation^29,33^ may then functionally derail microglia that disrupt synaptic functions^34–37^ and hijack cerebral homeostasis, potentially contributing to cognitive deficits.^27,37–39^ Compelling evidence highlights the significance of functional microbiome and the associated metabolites in controlling vagus nerve activity, to tonically transmit bidirectional signals from the viscera to the brain that regulate inflammation via neuronal motor efferents.^40–44^ Furthermore, microbiome-associated metabolites also tightly support the maintenance of gut-resident macrophages (gMacs),^45^ strategically situated in the muscularis externa, to interact with enteric neurons (ENs) that critically initiate gut motility.^46,47^ Hence, it is plausible to recognize the detrimental impact of pathogen-induced neuroinflammation on critically deteriorating host-brain homeostasis that may profoundly contribute to hypertauopathy and the consequences thereafter. Thus, considering pathogenic infection inciting neuroinflammation and how this can be reprogrammed through microbiome-associated metabolites serving as micronutrients and functioning as crucial factors that contribute to regulated genomic programs to potentially mitigate neurodegenerative diseases (e.g., AD) represent a novel line of inquiry and may warrant further investigations.^27^ To further delve into vitamin physiology, we posit that vitamin B12 controls the transcriptomic and metabolomic machinery of phagocytic cells (e.g., microglia, gMacs) and critically sustains healthy microbiomes and the associated metabolites to synergistically limit pathogen-induced neuroinflammation that may otherwise contribute to host memory loss.

Micronutrients play a pivotal role in maintaining overall health and facilitating optimal functioning of the human body. Among these, vitamin D is notable for its unique function as a potent immunomodulator. Sepsis is a complex and potentially life-threatening condition that stems from an excessive immune response to infection, resulting in organ dysfunction. Numerous studies have highlighted the potential correlation between inadequate vitamin D levels and an increased risk of sepsis among critically ill patients. However, clinical trials have shown that the early administration of high-dose enteral vitamin D3 during critical illness can rapidly correct vitamin D deficiency. Yet, this correction does not appear to yield discernible benefits in terms of mortality or other clinically significant outcomes,^48.49^ and the underlying reasons for this phenomenon remain elusive. The effects of vitamin D are primarily mediated through its receptor, known as the vitamin D receptor (VDR), which is distributed across various tissues in the body, including the intestines, kidneys, skin, immune cells, and different organ systems. VDR is known to exert immunomodulatory effects that influence the body’s response to infections and inflammatory processes.^50,51^ Vitamin D, in conjunction with VDR, can regulate the expression of numerous genes related to immune responses, impacting the microbiome/virome and host immunity.^52,53^ However, currently, there is limited information regarding the association between VDR and sepsis. Therefore, unraveling the intricate interplay among micronutrients, microbiome, and immune responses in the Veterans is crucial.

## Roadmap and gaps

**Figure 3.**
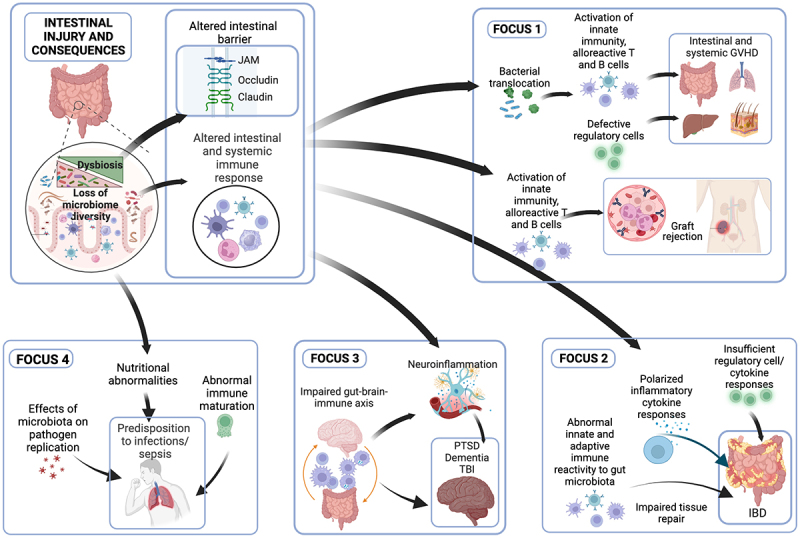
Focus areas of deployment-related diseases and their synergy in mechanistic studies for mechanisms and innovative treatments.

From meeting discussions, specific four Focuses are developed and tightly centered on defining translational and basic research platforms for reshaping the microbiome, detecting prognosticates, and treating prevalent chronic diseases in the Veteran population (Figure 3). Our central hypothesis was that dysregulation of the microbiome and immune interactions contributes to deployment-related diseases, including chronic intestinal, mental health, and neurologic disorders. An improved understanding of the microbiome and the relevance of critical micronutrients may provide in-depth insights into the underlying mechanisms implicated in the deficiency of these crucial cofactors and whether these vitamins can reprogram the molecular machinery of cells to resist pathogen-induced inflammation. The outcomes are anticipated to lead to the development of novel therapeutic interventions through pathways such as micronutrients, mitochondria, and microbiome functions.

**Figure 4.**
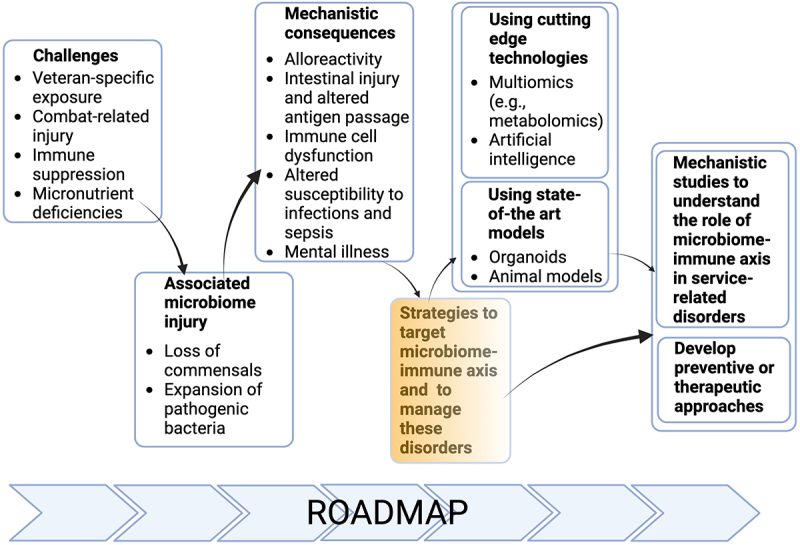
Roadmap of modulating microbiome-immune axis in the deployment-related chronic diseases of Veterans.

We agreed that for progress to be made, efforts should be directed to design mechanistic studies that address the interplay between the microbiome and immune system in pathogenesis of alloreactivity, intestinal and neuronal inflammation as well as local and systemic response to pathogen infection. Successful address of these mechanistic questions could benefit from the development and use of novel tools, such as organoids or novel preclinical animal models, or cutting-edge technologies, such as large-scale sample analysis using multiomics or application of artificial intelligence (AI) (Figure 4). To better understand the etiopathogenesis of these disorders in the context of microbiome-immune axis and to develop novel preventive or therapeutic approaches, we propose to develop and apply novel tools, such as organoids or humanized animal models, and cutting edge technologies, like spatial analysis, machine learning, multiomic approaches to microbiome and its metabolic activities. Of benefit will be the centralized and collaborative use of microbiome and metabolomic research, as well as the above-mentioned novel tools and cutting-edge technologies. To accomplish these goals and reach our strategic endpoints, we also propose to establish collaborative networks supported by efforts to centralize the data, share resources and develop core facilities/laboratories. Our efforts detailed on our roadmap are expected to help design novel mechanistic studies and develop novel therapeutic or preventative approaches to manage the clinical conditions discussed in four focus groups affecting Veterans.

The conference was based on delineating the commonalities and differences between conditions impacted by changes in regulated immunity and the microbiome and the associated metabolites in hosts. In response to the request for applications of collaborative merit review awards by the BLRD and CSRD of the Department of Veterans Affairs (RFA: BX23–007) and to investigate these commonalities and differences, we propose to submit several merit award applications, developed in synergy between focus groups despite the diversities among these groups, to address mutual aspects of service-related injuries and the consequences that may impact digestive physiology and extraintestinal organs.

## Conclusion and future plans

In summary, during this VA field-based meeting, we discussed specific diseases related to immunity and the microbiome. We identified four special focuses (Figure 3). Attendees started to establish the foundations of future research proposals during their presentations at the meeting, which highlighted the importance of mechanistic links between the microbiome and immune system, between the microbiome and diseases that the different focuses of the aforementioned research groups and between immune dysregulation and these diseases. At conclusion of the meeting, we developed a strategy on how to target these mechanistic links. The short-term goal is to develop a synergistic approach between grant applications before submission and structure innovative approaches that involve cutting edge technologies like machine learning to facilitate progress in this endeavor. In long-term goal, we are determined to characterize the functions of intestinal and extra-intestinal microbiomes, such as the oral microbiome, nasal microbiome, and lung microbiome, in health and disease, address-specific immune disorders prevalent in the Veteran population and the impact of dysbiosis on these diseases, and explore microbe-host immune interactions in the pathogenesis of these diseases and the role of microbiome/metabolite-based therapeutic approaches.

We expect our roadmap (Figure 4) to enable the researchers develop collaborative merit award applications in synergy to potentially identify novel and translatable therapeutic targets for the management of these disorders associated with deployment. This work could potentially be translatable to Veterans in other countries and more broadly to the worldwide population for disease prevention and treatment.

## Data Availability

No new data were generated during the meetings.
